# Discovering RNA-Based Regulatory Systems for *Yersinia* Virulence

**DOI:** 10.3389/fcimb.2018.00378

**Published:** 2018-10-25

**Authors:** Vanessa Knittel, Ines Vollmer, Marcel Volk, Petra Dersch

**Affiliations:** Department of Molecular Infection Biology, Helmholtz Centre for Infection Research, Braunschweig, Germany

**Keywords:** RNA thermometer, RNA stability, RNA processing, Csr/Rsm system, virulence, gene regulation, small regulatory RNAs

## Abstract

The genus *Yersinia* includes three human pathogenic species, *Yersinia pestis*, the causative agent of the bubonic and pneumonic plague, and enteric pathogens *Y. enterocolitica* and *Y. pseudotuberculosis* that cause a number of gut-associated diseases. Over the past years a large repertoire of RNA-based regulatory systems has been discovered in these pathogens using different RNA-seq based approaches. Among them are several conserved or species-specific RNA-binding proteins, regulatory and sensory RNAs as well as various RNA-degrading enzymes. Many of them were shown to control the expression of important virulence-relevant factors and have a very strong impact on *Yersinia* virulence. The precise targets, the molecular mechanism and their role for *Yersinia* pathogenicity is only known for a small subset of identified genus- or species-specific RNA-based control elements. However, the ongoing development of new RNA-seq based methods and data analysis methods to investigate the synthesis, composition, translation, decay, and modification of RNAs in the bacterial cell will help us to generate a more comprehensive view of *Yersinia* RNA biology in the near future.

## Introduction

**Figure 1:**
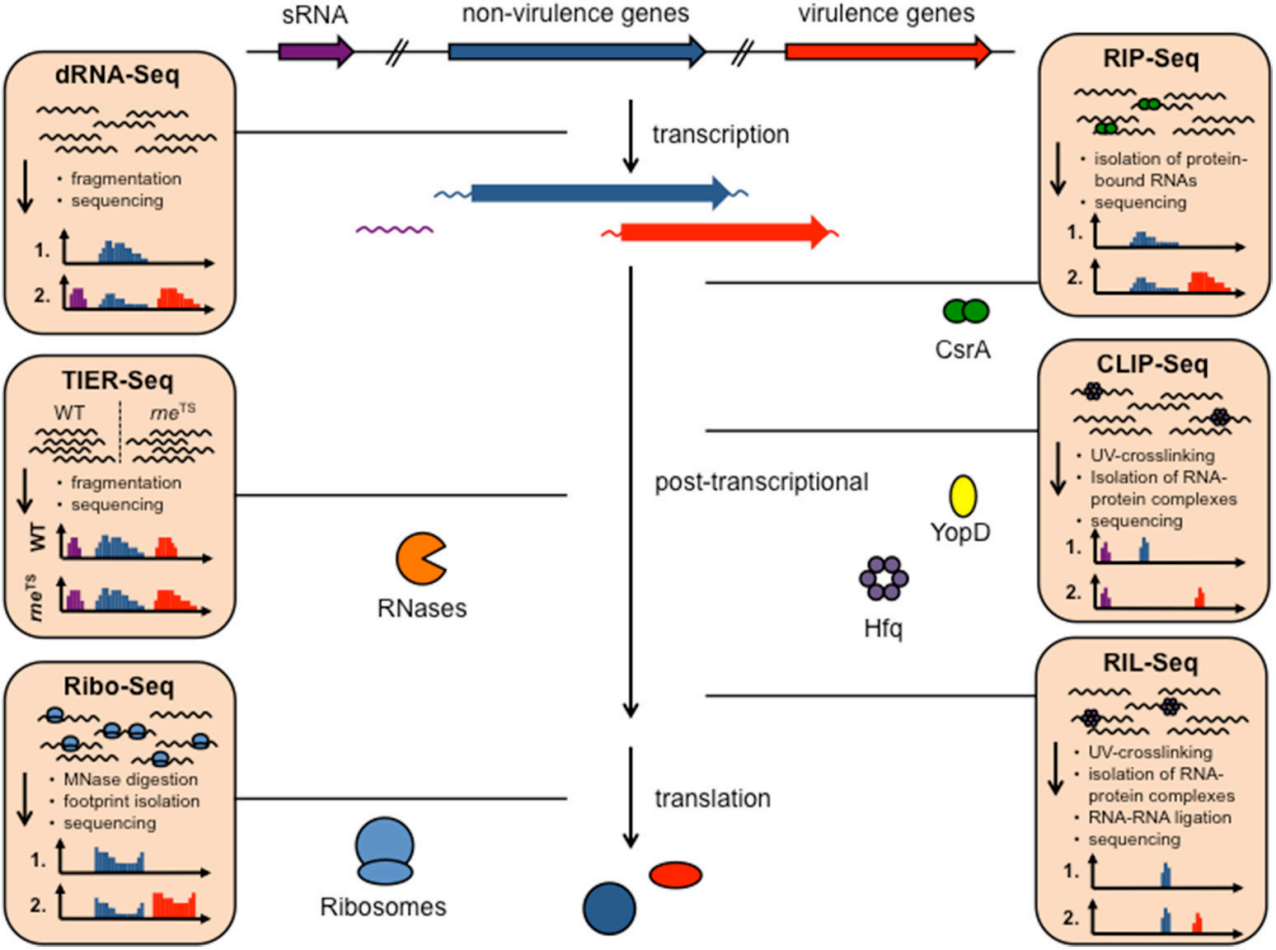
Available RNA-seq derived techniques, which can be used to study RNA-based control mechanisms in *Yersinia*. Different RNA-seq based techniques allow rapid and global investigation of transcript expression, transcription start sites, promoter structures, 5′- and 3′-UTR properties (dRNA-Seq), as well as binding of RNA-binding proteins to RNAs, such as CsrA, Hfq, and YopD (RIP-seq, CLIP-seq), RNA-RNA interactions facilitated by protein interaction (RIL-Seq), translation (Ribo-seq), and RNA processing and decay by heat-sensitive RNase E (*rne*^TS^) (TIER-seq).

For a long time it was thought that the RNA make-up and biology of bacteria is rather simple and constitutes mainly of mRNAs, tRNAs, and rRNAs and a few specific RNAs such as the tmRNA/SsrA of the ribosome rescue system. However, over the past 10 years, research on bacterial RNA molecules has proven this to be incorrect, since bacteria possess a huge variety of RNAs. A large repertoire of *cis*- and *trans*-acting non-coding RNAs has been identified and a plethora of different RNA-based regulatory mechanisms and functions has been unveiled mainly in *Enterobacteriaceae*, but lately also in many other prokaryotes. This showed that RNA-based gene expression control is highly complex and global, and almost as diverse and multifarious as transcriptional control. Multiple structured RNA elements have been identified in the 5′-untranslated regions of mRNAs sensing temperatures (RNA thermometers) or metabolites (riboswitches) in order to modulate mRNA translation and/or stability (Kortmann and Narberhaus, 2012; Sherwood and Henkin, 2016; McCown et al., 2017). Moreover, a remarkably large number of small regulatory RNAs (sRNAs) exist in bacteria (i.e., 200–300 in *Enterobacteriaceae*, Barquist and Vogel, 2015; Nuss et al., 2015), which control mRNA expression and decay often in concert with RNA-binding proteins. Among the most prominent RNA-binding proteins are the RNA chaperone Hfq and the carbon storage regulator (Csr) protein CsrA, as well as RNases. Clever variations of different global RNA-sequencing (RNA-seq)-based techniques (Figure 1) used to define the overall RNA-protein interactome and the sRNA-target networks demonstrated that almost 50% of the bacterial mRNAs are subjected to sRNA-mediated regulation (Melamed et al., 2016; Hör and Vogel, 2017; Waters et al., 2017).

The global analysis of RNA-based control strategies of virulence genes was started with a relatively small number of model bacteria. This included human pathogenic *Yersinia* species, such as *Y. pestis*, the causative agent of bubonic plague and the two enteric pathogens *Y. pseudotuberculosis* and *Y. enterocolitica* causing gut-associated diseases (Yersiniosis), i.e., ranging from enteritis, watery diarrhea, mesenteric lymphadenitis to post-infectious extraintestinal sequelae (Bottone, 1997; Koornhof et al., 1999; Smego et al., 1999; Valentin-Weigand et al., 2014). System level RNA-seq has the advantage that the entire transcriptome of an organism with all included RNA functions can be studied in a cellular and particular environmental (e.g., infection, virulence conditions) context. This allowed a single-nucleotide resolution and a dynamic view of the pathogen's gene expression, which are summarized and discussed in the present review. The establishment of comprehensive RNA maps of *Y. pseudotuberculosis* highlighted not only the breadth and large variety of RNA species, it also revealed new species-specific and conserved principles of RNA-based, post-transcriptional control strategies that contribute to the dynamic, spatial, and fine-tuned control of bacterial pathogenicity.

## Global transcriptional analysis to elucidate fitness-relevant traits of *Yersinia*

**Figure 2:**
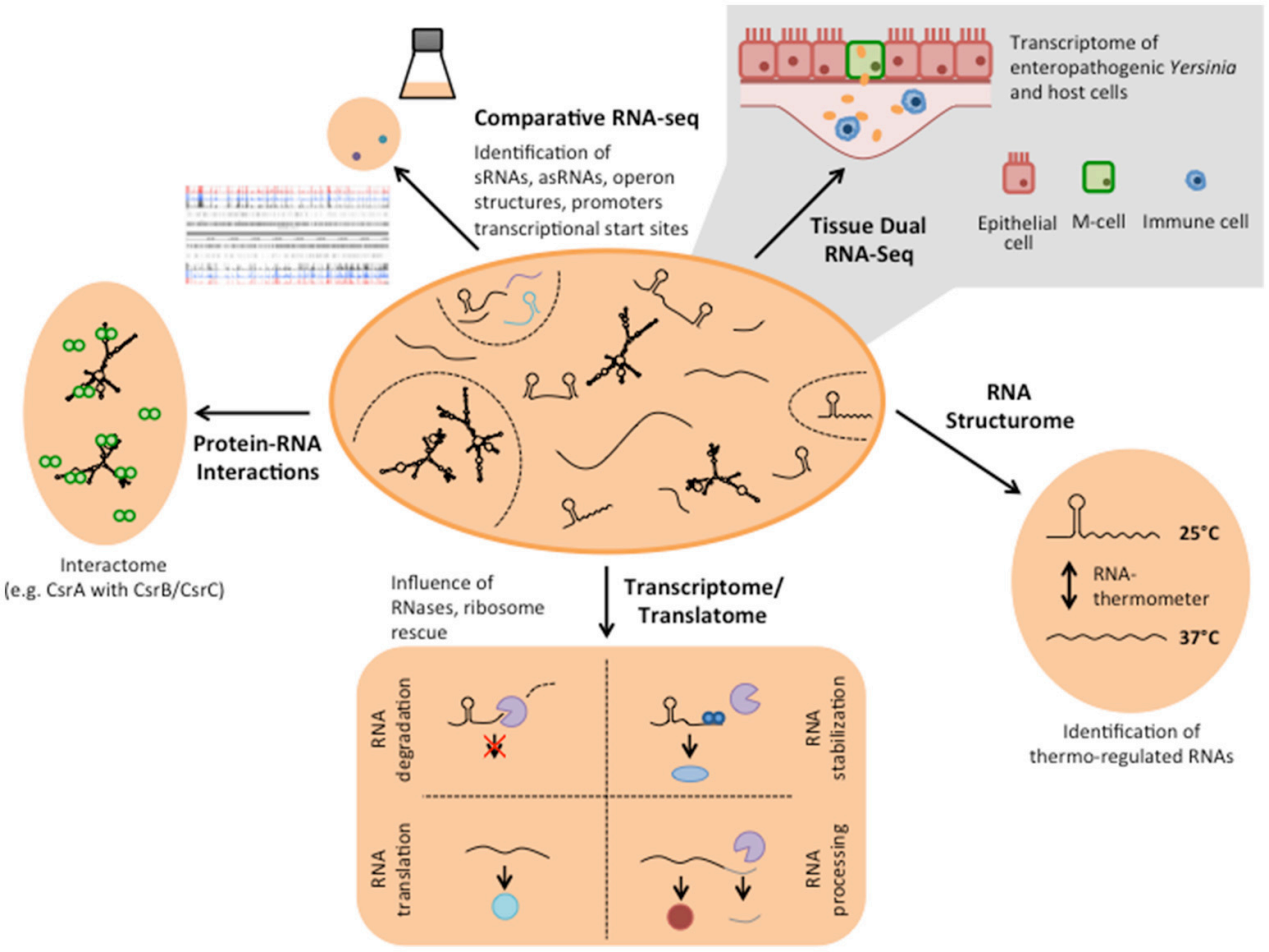
Different RNA-based regulatory mechanisms that control virulence in *Yersinia*. Global RNA biology approaches have been used to identify sRNAs, asRNAs, and sensory RNAs (RNA thermometer and riboswitches) as well as important RNA-binding proteins implicated in *Yersinia* virulence control.

In order to understand how the functional status of *Yersinia* is modulated in response to environmental conditions sensed outside or inside their hosts, detailed knowledge about the bacterial gene expression profile in different surroundings is required. RNA-seq is extremely well-suited for this task due to the high sensibility, high resolution and the possibility for high-throughput analysis of multiple *in vitro* and *in vivo* conditions (Kröger et al., 2013; Nuss et al., 2015, 2017a; Figures 1, 2). In particular transition from exponential to stationary phase and shifts from moderate temperatures to 37°C, mimicking host entry, were found to induce reprogramming of a large set of virulence genes. This includes colonization factors and immune defense mechanisms, encoded on the chromosome and the virulence plasmid, but also many catabolic/energy production genes. For instance, this uncovered the existence of a thermo-regulated “acetate switch,” which seems to prime the bacteria for growth in the digestive tract. This physiological switch occurs when bacteria shift from rapid growth in which they produce acetate from acetogenic carbon sources, e.g., glucose, to a metabolic program of slower growth, which is facilitated by the import and utilization of acetate (Nuss et al., 2015). This is accompanied by a thermo-induced up-regulation of multiple transport and catabolic genes for simple sugars. As the mammalian intestine is rich in short-chain fatty acids, in particular acetate, produced by the intestinal microbiota through consumption of available polysaccharides from diet and host-derived mucus (Cummings and Macfarlane, 1997), flipping the switch may facilitate ultilization of these microbiota-derived degradation products.

The RNA-seq approach also allowed us to elucidate the regulatory architecture linking nutritional status to virulence. We identified a massive remodeling of the CRP-controlled network in response to temperature and discovered CRP as a transcriptional master regulator of numerous conserved and newly identified non-coding RNAs which participate in this process (Nuss et al., 2015). This finding highlighted a new level of complexity of the regulatory network in *Yersinia*. The concerted action of several transcriptional regulators and multiple non-coding RNAs controlled by CRP adjusts *Yersinia* fitness and virulence to the requirements of their environmental and virulent life-styles. This discovery showed that CRP is an integral component of a regulatory network that controls life-style switching and highlighted the power of comparative RNA-seq analysis.

Parallel analysis of the transcriptomes of *Y. pseudotuberculosis* during mouse infection (Tissue Dual RNA-seq, Figure 2) has further allowed us to study host-pathogen interaction. It enabled us to correlate gene expression changes of the pathogen with those occurring in the host and revealed genes and regulatory RNAs that are predominantly expressed or repressed during the infection (Avican et al., 2015; Nuss et al., 2015, 2017a). Numerous alterations of host transcripts associated with IL-6-triggered inflammatory and acute phase responses (Mmp8 induction), coagulative activities, metal ion sequestration, tissue repair and damage highlighted that the immune response in the gut-associated lymphoid follicles (Peyer's patches) upon a *Y. pseudotuberculosis* infection is dominated by infiltration of neutrophils and dendritic cells, and elicits a mixed T_H_1/T_H_17 response (Nuss et al., 2017a; Heine et al., 2018). In response, *Yersinia* increases the gene and expression dose of the virulence plasmid-encoded Ysc type III secretion system (T3SS) and the anti-phagocytic Yop effector proteins (Yops) to prevent the phagocytic attack. In addition, the bacteria induce high-affine ion (Fe^2+^ and Zn^2+^) sequestration systems, diverse stress response, and nutrient uptake systems to counteract ion deprivation, radical/oxidative stress, anti-microbial peptides, and nutrient restraints (Nuss et al., 2017a). Tissue Dual RNA-seq further showed that the outcome of the observed pathogen-host interactions could vary significantly when expression of certain pathogenicity factors is reduced or abrogated. The cytotoxic necrotizing factor Y (CNF_Y_) expressed by some *Y. pseudotuberculosis* strains, e.g., YPIII and IP2666, is one of these factors. Suppression of CNF_Y_ triggers interferon-γ mediated responses fostering non-inflammatory bactericidal activities. Moreover, it promotes Ido1- and Arg1-mediated T_regs_ activation and proliferation and other immune suppressing and tolerogenic mechanisms that avoid systemic inflammation, prevent tissue destruction and drive this *Y. pseudotuberculosis* strain into persistency (Heine et al., 2018). This process is accompanied by a preterm reprogramming of the pathogen's transcriptome. Anaerobic and multiple acid and oxidative stress resistance genes (e.g., *arcA, fnr, frdA*, and *wrbA*) are already induced in the acute phase. It is assumed that this priming gives the bacteria a fitness edge against the host immune defense and facilitates establishment of a persistent microbiota-type life style (Heine et al., 2018). In parallel, the expression profile of many virulence genes changes toward a pattern seen at moderate temperature under *in vitro* growth conditions. Up-regulation of flagella and adhesion/invasion genes (e.g., *invA*) was observed during the establishment of persistence, whereas genes encoding important components of the T3SS and the Yops were down-regulated. This is accompanied by a change in the expression of the transcription factors of the Crp-CsrA-RovM-RovA regulatory cascade, indicating that these control factors determine the expression switch (Avican et al., 2015; Heine et al., 2018). Our findings suggest a life-style model for *Y. pseudotuberculosis* in which the bacteria change their gene expression profile from a virulent to an adapted phenotype, capable of persisting and spreading by fecal shedding. This further illustrates that the developed Tissue Dual RNA-seq approach is well suited to decipher the complexity of host-pathogen interactions during different stages of the infection. The comparative analysis between different strain isolates or mutants of potential virulence-relevant factors and regulators will help to decipher the complex interplay between of *Y. pseudotuberculosis* and the host response within distinct host niches.

## Annotation and characterization of *Yersinia* transcripts

Since RNA-seq provided a single-nucleotide resolution transcriptional landscape of *Y. pseudotuberculosis*, it was used to map all genes of the chromosome and virulence plasmid (Nuss et al., 2015, 2017a; Figure 2). This led to the identification of new, so far not annotated and previously overlooked open reading frames (ORFs) and revealed a large set of antisense RNAs and small *cis*- and *trans*-encoded regulatory RNAs (Koo et al., 2011; Nuss et al., 2015, 2017a). Some of the identified antisense RNAs are long and cover whole genes or multiple genes of the virulence plasmid (Nuss et al., 2015). However, their individual impact on gene expression is still unclear. Moreover, certain variations of the RNA-seq protocols (e.g., treatment of the RNA with the terminator 5′-phosphate-dependent exonuclease short TEX), led to an enrichment of primary transcripts with 5′-triphosphate ends. Comparative analysis between the transcriptomes obtained with or without TEX treatment, called differential RNA-seq (dRNA-seq, Sharma and Vogel, 2014, Figures 1, 2), allowed global mapping of the transcription start sites of all transcripts. This led to the identification of promoter elements and the definition of operon structures, and improved the annotation of the *Yersinia* genome and virulence plasmid (Nuss et al., 2015).

## The *Yersinia* RNA structurome

In contrast to double-stranded DNA, RNA is a single-stranded molecule. However, it can undergo significant intramolecular base-pairing to take in an individual three-dimensional structure. It is known for quite some time that the intrinsic structure of an RNA impacts many cellular processes, most frequently by modulating its translation and/or decay. Protein synthesis is mainly affected by RNA structures, which influence the accessibility of the ribosome binding site (RBS). Based on the whole-genome transcriptomics study of *Y. pseudotuberculosis* several potential *cis*-regulatory RNA elements have been identified in long 5′-untranslated regions (5′-UTRs; >200 nt) of virulence, stress adaptation and metabolic genes in *Y. pseudotuberculosis* (Nuss et al., 2015). A bioinformatics approach identified 19 riboswitch-like elements (RLEs) among the long 5′-UTRs. Riboswitches are complex RNA structures that undergo structural changes in response to binding of small molecules, typically metabolites or ions, altering the expression of their downstream gene on the transcriptional or post-transcriptional level (Breaker, 2011; Serganov and Nudler, 2013). These features make them ideal to sense small molecule concentrations, e.g., nutrient and ion availability. Five of these RLEs showed features homologous to the threonine operon leader, the Moco (Molybdenum cofactor biosynthesis), FMN and the cobalamin riboswitch of *E. coli* and a cation-responsive riboswitch upstream of an Mg^2+^ transporter gene (*corA*) similar to *mgtA* of *Salmonella* (Nuss et al., 2015). However, the function of the majority of the riboswitch-like elements remains uncovered.

Structural alteration of a sensory RNA segment can also be triggered by temperature shifts. For instance, a thermo-controlled melting process of a double-stranded region, like a molecular zipper, is used to modulate gene expression of several bacterial heat shock and virulence-relevant genes (Kortmann and Narberhaus, 2012). One of these thermo-sensitive RNA elements (RNA thermometer) was identified upstream of *lcrF* (Table 1), the virulence plasmid-encoding gene for the master regulator of the T3SS/Yop machinery. The *lcrF* RNA thermometer is highly conserved between the human pathogenic *Yersinia* species and consists of a two stem-loop structure of which the second hairpin sequesters and hides the ribosome binding site (RBS) within its stem region at moderate temperatures (20–30°C). Elevated temperatures (37°C) lead to partial unfolding of the stem-loop, which renders the RBS accessible for the small subunit of the ribosome, leading to translation initiation (Hoe and Goguen, 1993; Böhme et al., 2012).

**Table 1:** RNA regulators of *Yersinia* important for virulence.

**Regulators**	**Mechanisms/Targets**	**Virulence-associated processes**	**Regulation**	**References**
***Trans*****-ENCODED ncRNA**				
CsrB/CsrC	Small structured RNAs with multiple GGA sequences that bind and sequester CsrA, Hfq-dependent	Control of RovM, RovA, InvA/PsaA adhesins, T3SS/Yops, host-adapted metabolism, motility, carbon metabolism, stress resistance	Controlled by BarA/UvrY, PhoP/PhoQ, Crp, short chain fatty acids, acidic pH, antimicrobial peptides	(Heroven et al., 2008, 2012; Romeo et al., 2013; Bücker et al., 2014; LeGrand et al., 2015; Kusmierek and Dersch, 2017; Ozturk et al., 2017)
RybB	Hfq-dependent sRNA	Induced in the lung and spleen during infection with *Y. pestis* biovar microtus	Temperature and growth phase-dependent	(Koo et al., 2011; Yan et al., 2013; Nuss et al., 2015, 2017a)
RyhB1/RyhB2	Homologous Hfq-dependent sRNAs	*ryhB1* and *ryhB2* have a small influence on virulence in *Y. pestis* biovar microtus and *Y. pseudotuberculosis*	Increased during iron starvation, induced in the lung and spleen during infection with *Y. pestis* biovar microtus, and induced in Peyer's patches of mice, they depend on growth phase, they are controlled by the regulator Fur, degraded by PNPase	(Deng et al., 2012, 2014; Yan et al., 2013; Nuss et al., 2017a)
GlmZ/GlmY	Homologous sRNAs, GlmZ activates *glmS* mRNA translation by an anti-antisense mechanism, GlmY acts upstream of GlmZ and positively regulates *glmS* by antagonizing GlmZ RNA inactivation	Control amino-sugar metabolism, GlmY/GlmZ control *glmS*, which encodes the enzyme glutamine synthase necessary for the synthesis of *N*-acetylglucosamine-6-P, which is used for cell wall biosynthesis	Regulated by RNase E and Hfq	(Görke and Vogel, 2008; Urban and Vogel, 2008; Nuss et al., 2017a)
SsrA/tmRNA	A-site of stalled ribosomes, binds together with SmpB to stalled ribosomes by mimicking a tRNA and mRNA, which replaces incomplete/truncated transcripts within stalled ribosomes	Rescues stalled ribosomes, holds the translation machinery in the operation mode	Induced in the lung and spleen during infection with *Y. pestis* biovar microtus	(Okan et al., 2006, 2010; Yan et al., 2013)
SgrS	SgrS activates synthesis of the phosphatase YigL in a translation-independent fashion the SgrS RNA targets the *pldB* mRNA and blocks sustained 5'- to 3'- endonucleolytic turnover of the *pldB*-*yigL* transcript by RNase E	Phospho sugar stress, SgrS-mediated increase of phosphatase YigL leads to the dephosphorylation of the accumulated sugars and facilitates their export by efflux systems	Glucose-6-P-responsive, accumulation of phospho sugars is toxic and activates SgrS RNA through the SgrR transcription factor	(Vanderpool and Gottesman, 2004; Papenfort et al., 2012, 2013; Bobrovskyy and Vanderpool, 2014, 2016; Nuss et al., 2017a)
Ysr29	–	General stress response (GroEL, DnaK, UreC, S/RpsA, Gst, AhpC, Rrf), required for stress response and full virulence of *Y. pseudotuberculosis*	Temperature and growth phase-dependent	(Koo et al., 2011)
Ysr35	–	Required for full virulence of *Y. pseudotuberculosis* and *Y. pestis*, controls several general stress response factors such as GroEL, DnaK, the peroxidase AhpC and the translation factors RpsA and Rrf	Temperature-induced	(Koo et al., 2011)
Ysr141	–	Influences expression and secretion of T3SS/Yop components and the major regulator LcrF, modulates host immune defense, has direct influence on YopJ translation	–	(Schiano et al., 2014)
Ysr170	–	Important for intracellular replication of *Y. pestis* in cultured macrophages	–	(Li et al., 2016)
**ANTISENSE RNA**				
CopA	Complementary to the replicase gene *repA* of the *Yersinia* virulence plasmid	Repression of the replication of the virulence plasmid pYV, repression of *rep*A mRNA translation/stability, reduces expression of the T3SS/Yop components	Downregulated during colonization of the Peyer's patches	(Qu et al., 2012; Wang et al., 2016)
**RNA THERMOMETER**				
*Ail*	5′-UTR of the adhesin gene *ail*	Stem-loop structure restricts access of ribosomes to the ribosome binding site at 25°C but not at 37°C, regulation of the expression of the cell attachment and invasion outer membrane protein Ail	Temperature-induced	(Rhigetti et al., 2016)
*cnfY*	5′-UTR of the toxin gene *cnfY*	A stem-loop structure restricts access of ribosomes to the ribosome binding site at 25°C but not at 37°C, regulation of the expression of the cytotoxic necrotizing factor CNF_Y_	Temperature-induced, controlled by *csrA, crp*	(Schweer et al., 2013; Rhigetti et al., 2016)
*lcrF*	*ysw-lcrF* intergenic region, FourU RNA thermometer	A two stem-loop structure restricts access of ribosome to the ribosome binding site at 25°C but not at 37°C, proper function required for expression of the T3SS/*yop* genes and virulence	Temperature-induced, iron limitation, and oxidative stress, controlled by the transcription factors YmoA, RcsB, IscR	(Hoe and Goguen, 1993; Böhme et al., 2012; Schwiesow et al., 2015; Rhigetti et al., 2016)
*katA*	5′-UTR of the katalase gene *katA*	Resistance against oxidative stress	Thermally induced structural changes liberate the ribosomal binding site, induced by oxidative stress	(Rhigetti et al., 2016)
*sodA*	5′-UTR of the superoxide dismutase gene *sodA*	Resistance against oxidative stress	Thermally induced structural changes liberate the ribosomal binding site, induced by oxidative stress	(Rhigetti et al., 2016)
*sodB*	5′-UTR of the superoxide dismutase gene *sodB*	Resistance against oxidative stress	Thermally induced structural changes liberate the ribosomal binding site, induced by oxidative stress	(Rhigetti et al., 2016)
*sodC*	5′-UTR of the superoxide dismutase gene *sodC*	Resistance against oxidative stress	Thermally induced structural changes liberate the ribosomal binding site, induced by oxidative stress	(Rhigetti et al., 2016)
**RIBOSWITCH**				
*mgtA/corA*	Mg^2+^ binding RNA secondary structure in the 5′-UTR of the Mg^2+^ transporter gene *mgtA*, Mg^2+^ binding initiates early Rho-independent termination of *mgtA* transcription through conformational changes in the RNA	This riboswitch regulates Mg^2+^ uptake, essential for survival and replication of macrophages	*mgtA* expression is induced under high Mg^2+^ concentrations	(Korth and Sigel, 2012; Nuss et al., 2015)
**RNA-BINDING PROTEINS**				
CsrA	CsrA is a global RNA binding protein of the carbon storage regulator system. It interacts with single-stranded GGA motifs within stem-loop structures of mRNAs or the regulatory RNAs CsrB and CsrC, and modulates translation efficiency and stability of mRNAs and regulatory RNAs	CsrA controls multiple virulence- and fitness-relevant traits, e.g., motility, adhesion and invasion factors (YadA, InvA, PsaA), T3SS/Yops, regulatory proteins such as RovM and RovA, various metabolic functions (carbon metabolism), resistance against environmental stresses	CsrA function is controlled by the regulatory RNAs CsrB and CsrC, induced during stationary phase	(Heroven et al., 2008, 2012; Bücker et al., 2014; LeGrand et al., 2015; Willias et al., 2015)
Hfq	Hfq is a global RNA binding protein, preferential binding to AU-rich motifs, interacts with multiple regulatory RNAs and mRNAs, Hfq acts as an RNA chaperone, which enhances and stabilizes interaction of regulatory RNAs with target mRNAs	Loss of the *hfq* gene affects multiple virulence-related traits, e.g. expression of outer membrane adhesins, biofilm formation and cyclic-di-GMP levels, lipid A structure, outer membrane vesicle synthesis, and motility	Induced during stationary phase, dependent on temperature	(Geng et al., 2009; Schiano et al., 2010; Bellows et al., 2012; Rempe et al., 2012; Schiano and Lathem, 2012; Eddy et al., 2014; Kakoschke et al., 2014, 2016; Nuss et al., 2015; Leskinen et al., 2017)
SmpB	SmpB is a specific RNA binding protein that interacts with the A site of ribosomes together with SsrA, SmpB assists SsrA interaction with stalled ribosomes to rescue the translation machinery on mRNAs from truncated transcripts without a stop codon	The SmpB/SsrA system influences *ysc/yop* expression and type III secretion, affects resistance against environmental stresses experienced within phagocytic cells (e.g., oxidative, nitrosative and acidic stress)	Upregulated during infection with *Y. pestis* biovar Microtus in the lungs	(Okan et al., 2006, 2010)
YopD	RNA-binding protein, translocator protein, interaction partner of the chaperone LcrH and the protein LcrQ (YscM1 and YscM2 in *Y. enterocolitica*), interacts with 5′-UTRs of *ysc/yop* mRNAs, binds to ribosomes, RNA-binding mechanism and ribosomal interaction partner are unknown	Influences expression of the *ysc/yop* genes, Ca^2+^-blind/independent expression of the T3SS	LcrF-dependent expression, temperature-regulated, host cell contact-induced	(Williams and Straley, 1998; Anderson et al., 2002; Cambronne and Schneewind, 2002; Chen and Anderson, 2011)
**RNases**				
RNase E	RNA degradation, endonuclease, cleaves RNA substrates in single-stranded regions followed by a stable stem-loop structure, RNase E is part of the degradosome, a multiprotein complex that includes PNPase	RNase E influences secretion of the T3SS effectors	–	(Yang et al., 2008)
PNPase	RNA degradation, exonuclease, cleaves RNA substrates from the 5'- and 3'-end. PNPase is part of the degradosome, a multiprotein complex, and cooperates with RNase E	PNPase influences secretion of the T3SS effectors, influences resistance against oxidative stress and growth in the cold	–	(Rosenzweig et al., 2005, 2007; Henry et al., 2012; Rosenzweig and Chopra, 2013)
YbeY	RNA decay, processing of 3′-ends of the 16S rRNA, responsible for the late stage 70S ribosome quality control	Pleiotropic, controls many virulence-relevant traits, including acid stress resistance, cell adhesion/invasion properties and T3SS, controls regulatory RNAs CsrB and CsrC	–	(Leskinen et al., 2015)
RNase III	RNA decay, binds and cleaves double-stranded RNA, processing of ribosomal RNA precursors and of some mRNAs	Affects abundance of the RyhB2 transcript	–	(Deng et al., 2014)

To investigate whether additional RNA thermometers or thermo-sensitive RNA elements are encoded on the *Y. pseudotuberculosis* genome, a structure RNA profile was recently generated of an entire transcriptome (Figure 2). For this purpose total RNA of the bacteria were isolated, incubated at 25, 37, and 42°C, and subsequently treated with single-strand and double-strand specific RNases. RNA-seq analysis of the resulting RNA fragments (PARS: parallel analysis of RNA structures) from 1,750 transcripts identified numerous temperature-sensitive RNAs (Rhigetti et al., 2016). This revealed that the RNA structurome of this pathogen is highly dynamic and encodes many more RNA thermometers. In total, 16 new RNA thermometers were discovered in 5′-UTRs of metabolic, heat stress, and virulence-relevant genes. Among them are many oxidative stress genes (*sodA, sodB, sodC*, and *katA*), and two very important virulence genes, encoding the cell adhesion and invasion factor Ail, contributing to host cell colonization and host resistance, and the CNF_Y_ toxin, triggering host tissue inflammation and damage (Rhigetti et al., 2016; Table 1). Besides the putative RNA thermometer, several small regulatory RNAs and tRNAs were also shown to be thermo-sensitive in this study. It will be interesting to investigate whether they promote and/or participate in the thermo-regulation of their target mRNA(s), and whether they are exploited by the pathogen to modify bacterial metabolism, physiology or pathogenicity upon entering a warm-blooded host, similar to the RNA thermometers.

## Control by regulatory RNAs

Small regulatory RNAs (sRNAs) are central regulators that play an essential role in various physiological and virulence associated processes in bacteria (Papenfort and Vogel, 2010, 2014; Heroven et al., 2017; Quereda and Cossart, 2017; Westermann, 2018). sRNAs are involved in the fine-tuning of these processes, allowing a rapid adaptation of pathogens to new environments. They have unique regulatory characteristics and control target gene expression post-transcriptionally through base-pairing with their transcripts. Such interaction can alter translation initiation or mRNA stability (Fröhlich and Vogel, 2009; Storz et al., 2011; Gorski et al., 2017). Functional analysis of sRNAs in different microorganisms revealed that often several sRNAs are part of the same regulatory cascade and sRNA-target network. This network typically includes multiple feedback loops and mixed regulatory circuits, illustrating the true complexity of RNA-based control system (Nitzan et al., 2017; Hör et al., 2018).

A myriad of sRNAs have been identified in the human pathogenic *Yersinia* species in independent high-throughput RNA-seq studies and are mainly referred to as Ysr (*Yersinia* small RNAs) (Koo et al., 2011; Koo and Lathem, 2012; Qu et al., 2012; Beauregard et al., 2013; Yan et al., 2013; Nuss et al., 2015, 2017a). The Ysrs are presumed *Yersinia*-specific sRNAs that were identified in an sRNA search in *Y. pseudotuberculosis* (Koo et al., 2011). A detailed list and the characteristics of these sRNAs are summarized in two recent reviews (Martínez-Chavarría and Vadyvaloo, 2015; Nuss et al., 2017b) (Figure 2, Table 1). Two aspects that are particularly striking are that (i) many sRNAs are directly regulated by the cAMP receptor protein (Crp) in response to growth phase and temperature, and that (ii) the majority of sRNAs are specific for *Yersinia*. This indicates that they are important players for the biological fitness and adaptation in their individual environmental and host niches, and reservoirs. A drawback of the latter feature is that most bioinformatic algorithms to identify potential targets of sRNAs rely on comparative genomics, i.e., functional and network analysis depending on sequence conservation between species (Wright et al., 2013), and are thus not suitable for the analysis of most Ysr sRNAs. Consequently, very little information is available about their molecular function and role for the pathogen. Nonetheless, the RNA chaperone Hfq, which mediates annealing of many sRNAs to their target mRNAs and protects them from ribonuclease cleavage or facilitates sRNA turnover or activity, was found to be required for virulence (Table 1). An *hfq* mutant of *Y. pestis* is impaired in its ability to resist phagocytosis and survive within macrophages at the initial stage of infection, and is highly attenuated in mice after subcutaneous or intravenous injection (Geng et al., 2009). Moreover, Hfq was found to regulate biofilm gut blockage that facilitates flea-borne transmission of *Y. pestis* (Rempe et al., 2012). Hfq of *Y. pseudotuberculosis* and *Y. enterocolitica* modulates the expression of multiple virulence-relevant cell surface structures such as adhesins/invasins (e.g., Ail, OmpX, MyfA pili, YadA, InvA) and expression of the T3SS/*yop* genes (Schiano et al., 2010, 2014; Schiano and Lathem, 2012; Kakoschke et al., 2014, 2016). Moreover, several Hfq-dependent alterations in the lipid A structure, motility, and biofilm formation defects, and changes in outer membrane vesicle synthesis were identified, which also impact virulence (Eddy et al., 2014; Leskinen et al., 2017). This strongly indicates that Hfq acts by controlling the expression of virulence-associated genes, probably in conjunction with small non-coding RNAs. However, up to now only very little is known about virulence-associated sRNAs and whether they are Hfq-dependent.

An initial analysis with a few Ysr sRNAs showed that Ysr29, Ysr35, and Ysr141 seem to play a role in pathogenesis (Koo et al., 2011; Schiano et al., 2014; Table 1). The specific target of Ysr35 remains unknown, but expression of multiple proteins was found to be affected in an *ysr35* mutant. Among them are several general stress response factors such as the chaperones GroEL and DnaK, the peroxidase AhpC, the translational factors S1 (RpsA) and the ribosome recycling factor Rrf (Koo et al., 2011). The virulence plasmid-encoded sRNA Ysr141 was found to enhance the synthesis of LcrF and many effector proteins (YopJ, YopE, YopK, YpkA), whereby only a direct influence of Ysr141 on YopJ translation was documented (Schiano et al., 2014). Recent work has also identified multiple sRNAs of *Y. pestis*, which are upregulated within macrophages. Among them is Ysr170, which was shown to be important for intracellular replication (Li et al., 2016). Much more is known about conserved sRNAs and their impact on pathogenicity of *Yersinia*. The most prominent influence on pathogenesis has been shown for the global carbon storage regulator system (Csr) (Heroven et al., 2012; Romeo et al., 2013; Vakulskas et al., 2015; Kusmierek and Dersch, 2017; Nuss et al., 2017b). The Csr system of *Yersinia* consists of the small RNA-binding protein CsrA, which is highly conserved among many bacterial species, and the two non-coding sRNAs CsrB and CsrC. The CsrA protein typically affects the translation and/or stability of target mRNAs by binding to GGA motifs within the mRNA. Activity of CsrA is controlled and antagonized by the sRNAs CsrB and CsrC as these are able to sequester multiple CsrA molecules, which prevents binding of target transcripts (Romeo et al., 1993, 2013; Liu and Romeo, 1997; Liu et al., 1997; Romeo, 1998; Babitzke and Romeo, 2007; Romeo and Babitzke, 2018). In *Yersinia*, similar to other *Enterobacteriaceae*, the Csr system constitutes an important link between metabolic, stress, and virulence gene regulation by sRNAs, and plays a crucial role in virulence (Heroven et al., 2012; Kusmierek and Dersch, 2017; Nuss et al., 2017b). It has been found to control expression of the flagella master regulator FlhDC, the colonization factors InvA and PsaA, and multiple fitness and virulence-relevant cellular processes. These are e.g., motility/chemotaxis, biofilm formation, resistance against various stresses, including antibiotics and oxygen radicals, and adaptation of the central carbon metabolism by regulating the pyruvate-acetyl-CoA-tricarboxylate acid cycle flux (Heroven et al., 2008; Bücker et al., 2014; LeGrand et al., 2015; Willias et al., 2015; Kusmierek and Dersch, 2017). A very recent study of our group further demonstrated that the abundance of both CsrB and CsrC sRNAs in *Y. pseudotuberculosis* is reduced during the colonization of Peyer's patches compared to growth under different *in vitro* conditions (Nuss et al., 2017a). This indicates that more “free” CsrA is required during the infection process. In fact, loss of CsrA was shown to reduce the synthesis and completely abolish the secretion of the Yop effector proteins in *Y. pseudotuberculosis* (Nuss et al., 2017a). Most interestingly, CsrA also influences Yop secretion in *Y. enterocolitica* (Ozturk et al., 2017), although the overall outcome is quite different. This might be explained by the different growth conditions used for the Yop secretion assay or differences between the species.

Furthermore, RNA-seq approaches used to profile gene expression of *Y. pseudotuberculosis* and *Y. pestis* during host colonization revealed several other conserved sRNAs that are induced during infection. Among these sRNAs are RyhB1 and RyhB2—two sRNAs that are induced during iron starvation experienced in host tissue, SgrS—an sRNA upregulated under glucose-phosphate stress, and GlmY involved in cell wall synthesis (Table 1) (Massé and Gottesman, 2002; Vanderpool and Gottesman, 2004; Reichenbach et al., 2008; Urban and Vogel, 2008; Wadler and Vanderpool, 2009; Deng et al., 2012; Papenfort et al., 2012, 2013; Yan et al., 2013; Bobrovskyy and Vanderpool, 2014, 2016; Nuss et al., 2017a). However, lack of these sRNAs had only a mild effect on host colonization by the pathogens, indicating that they are implicated in the fine-tuning of virulence-relevant fitness processes.

Of the 80 antisense RNAs (asRNAs) that have been discovered to be part of the *Y. pseudotuberculosis* transcriptome, multiple are encoded on the virulence plasmid. Several were complementary to transcripts of the T3SS/Yop genes, although their influence on the expression of these virulence genes, and the influence on *Yersinia* virulence has not yet been investigated. However, one asRNA, CopA/*incRNA*, has been shown to be extremely important for the virulence process. CopA is implicated in the RNA-based control of the copy number of the virulence plasmid. The copy number of the *Yersinia* virulence plasmid, an IncFII plasmid, is controlled by the replicase RepA, which is tightly controlled and negatively regulated at the transcriptional and the post-transcriptional level. Under non-secretion conditions (e.g., in the absence of host cells), synthesis of RepA is repressed by (i) CopB, a transcriptional repressor that interacts with the *repA* promoter, and (ii) the asRNA CopA, that binds to the 5′ end of the longer of two *repA* transcripts preventing translation of a short leader peptide whose translation is coupled with and required for *repA* translation (Blomberg et al., 1990, 1992; Nordström, 2006; Wang et al., 2016; Pilla and Tang, 2018). Despite other IncFII plasmids, transcription of *copA* and *copB* of the *Yersinia* virulence plasmid are not constitutive. They are temperature-regulated and repressed during infection of the Peyer's patches, leading to a reduction of the ratio of the CopA asRNA and the *repA* transcript level, which results in an increase from 1–4 to up to 4–12 plasmid copies per bacterial cell during host tissue colonization (Wang et al., 2016). It could be demonstrated that up-regulation of the virulence plasmid copy number during the infection is crucial for virulence and that one of the secreted T3SS substrates (YopD) is involved. However, the molecular mechanism coupling copy number control to host cell binding still needs to be elucidated.

In summary, the identification of regulatory sRNAs and asRNAs using RNA-seq in the context of an infection process highlights that this approach is a powerful tool for the discovery of new infection-relevant regulatory processes. Application of newly developed RNA-seq technologies such as pulse-chase-expression global target searches (Massé et al., 2005; Papenfort et al., 2006; Westermann et al., 2016), coupling of RNA-seq with ribosome profiling (Guo et al., 2014; Wang et al., 2015), M2-tagged sRNA affinity purification coupled with RNA-seq (Lalaouna et al., 2015; Tomasini et al., 2017), RNA interaction by ligation and sequencing—short RIL-Seq (Melamed et al., 2016), or global small non-coding RNA target identification by ligation and sequencing—short GRIL-seq (Han et al., 2016) could help to identify direct targets of the regulatory RNAs and their role in *Yersinia* biology.

## Translational control

During translation mRNA is decoded for protein synthesis in a multi-step process consisting of initiation, elongation, termination, and recycling (Melnikov et al., 2012). This process is performed by the ribosome and several other translation associated components like tRNAs, initiation factors (IF), elongation factors (EF), and recycling factors (RF and RRF) (Melnikov et al., 2012). In addition to the complex transcriptional control strategies, gene expression of *Yersinia* is extensively regulated on the level of translation. Regulation of translation initiation occurs by changing the accessibility of the RBS for the small ribosomal subunit (30S, small subunit). As previously described, this can be achieved by masking the RBS with an RNA binding protein such as CsrA (Heroven et al., 2008, 2012) or an RNA thermo-loop (Baba et al., 1991; Böhme et al., 2012).

Other important riboregulators can modulate sRNA-mRNA interaction and influence the function of the translation machinery. For instance, the *trans*-translation control system or ribosome rescue system is composed of a small RNA-binding protein SsrB which transfers the polypeptide chain of ribosomes that are stalled on damaged or incomplete transcripts to the small, stable sRNA SsrA/tmRNA (Okan et al., 2006). The fact that SsrA/tmRNA is strongly upregulated in *Y. pestis* during the colonization of the lung and spleen and an *ssrA* mutant strain is strongly attenuated for virulence in a mouse infection model, strongly indicated that ribosome rescue is crucial for virulence (Okan et al., 2010; Yan et al., 2013). Reduced stress resistance and influence of the expression of the major virulence regulator LcrF in an *ssrA/ssrB* mutant strain could be the reason for this drastic phenotype (Okan et al., 2006, 2010).

One additional and very special translational control pathway of *Yersinia* virulence is mediated by the T3SS protein YopD, which, together with YopB, builds up the translocon pore in the eukaryotic membrane. Under non-secretion conditions, YopD is present in the bacterial cell and interacts with its chaperone LcrH, which prevents its degradation (Francis and Wolf-Watz, 1998; Edgren et al., 2012; Dewoody et al., 2013). Alone or bound to its chaperone LcrH, YopD seems to use sequences within the AU-rich regions in the proximity of the RBS of multiple *yop*-encoding mRNAs. This hinders translation initiation and reduces Yop protein expression (Williams and Straley, 1998; Anderson et al., 2002; Chen and Anderson, 2011). However, other control factors seem to contribute to YopD-mediated translational repression, as the identified 5′-UTR regions are important, but do not seem to be sufficient to promote this process. In fact, the secreted factor LcrQ (LcrM1 and LcrM2 in *Y. enterocolitca*) was found to contribute to post-transcriptional repression by YopD (Cambronne and Schneewind, 2002). Moreover, YopD was found to interact with the small 30S subunits of the ribosome. This indicates the presence of a different population of ribosomes (ribosome heterogeneity, Byrgazov et al., 2013), which negatively influences translation initiation complex formation (Kopaskie et al., 2013). How and to what extent these interactions affect translation of virulence-encoded genes is still unknown. YopD-binding could block the anti-RBS in the 16S rRNA from binding with the RBS. However, this alone would not explain why in particular translation of *yop* mRNAs is abrogated.

## RNA processing and decay

Fast adaptation to changing surroundings is crucial for bacterial pathogens, such as *Yersinia* cycling between various environmental reservoirs, mammals, and humans in order to efficiently colonize their respective host and thrive in an occupied niche. Essential for this process is a controlled degradation of transcripts, which is mediated by a complex set of ribonucleases (RNases). The amount and type of RNases, their essentiality and collaboration in RNA metabolism is strongly depending on the investigated pathogen (e.g., *Yersinia pseudotuberculosis* > 15 RNases) (Arraiano et al., 2010; Lawal et al., 2011; Hui et al., 2014). The different RNases vary to a great extend in their mode of action, specificity for certain RNA features, and the nature of their targets in general (Arraiano et al., 2010; Lawal et al., 2011; Hui et al., 2014). Most bacteria, including all human pathogenic *Yersinia* species, contain low-specificity single-stranded endonucleases (e.g., RNase E), double-stranded endonucleases (e.g., RNAse III) and 3′-exonucleases (e.g., PNPase) (Mohanty and Kushner, 2016). RNA degradation occurs typically by consecutive endonucleolytic cleavage followed by exonucleolytic degradation from the 3′-end. The 5′-end is generally more stabilized due to the triphosphate end, but also the 3′-end can be rendered more stable toward RNases, e.g., by the presence of hairpin structures, RNA modification/polyadenylation or coverage by translating ribosomes. In addition to maturation and activation of certain transcripts (e.g., rRNA, tRNA), several conserved RNases were found to play a crucial role in global and/or targeted mRNA and sRNA turnover and also virulence in many pathogens (Clements et al., 2002; Ygberg et al., 2006; Lawal et al., 2011; Rosenzweig and Chopra, 2013).

In order to determine global mRNA decay rates, the bacterial culture is generally treated with the RNA polymerase inhibitor rifampicin prior to isolation of total RNA. To identify RNase targets and decay pathways, RNA-seq approaches with a transiently inactivated endoribonuclease followed by RNA-seq—short TIER-seq (Chao et al., 2017, Figure 1) or with RNase-deficient mutants were recently used to quantify transcript steady-state levels in Leskinen et al. (2015) and Chen et al. (2016).

In *Yersinia*, especially the RNases PNPase and RNase E were found to occupy well-established roles in the regulation of virulence factors and growth under several infection-relevant conditions (oxidative and cold stress) (Rosenzweig et al., 2007; Yang et al., 2008; Rosenzweig and Chopra, 2013). PNPase forms together with the RNase E, the helicase RhlB and the glycolytic enzyme enolase a multi protein complex, called the degradosome (Rice and Vanderpool, 2011). RNase E represents the scaffolding enzyme for this complex hyperstructure and while some of the affected virulence-relevant properties were associated with an intact buildup of the degradosome, others only require RNase E or PNPase activity and RNA binding ability (Henry et al., 2012; Rosenzweig and Chopra, 2013). A *Yersinia pnp* mutant is significantly less virulent in the mouse compared to the isogenic wild-type strain. It was shown that PNPase is required for optimal functioning of the T3SS. Yet, this did not seem to depend on its ribonuclease activity, but requires its S1 domain. As the *pnp* mutant strain also expressed similar or even enhanced levels of T3SS-encoded transcripts, it is most likely that its virulence attenuation is due to a limiting T3SS activity (Rosenzweig et al., 2005, 2007). Besides the RNases of the degradosome, there is an increasing amount of information that additional RNases contribute to virulence. For instance, the single-strand specific RNase YbeY, which is important for the processing of the 3′-end of the 16S rRNAs, was recently shown to influence the expression of many virulence genes (colonization factors, T3SS, and the amount of both Csr regulatory RNAs) (Leskinen et al., 2015). How precisely YbeY influences all these processes is still unclear, but the large number of differentially regulated fitness and virulence-relevant genes in the YbeY-deficient mutant correlates with the severe phenotype and illustrates a crucial role in cell homeostasis and virulence. Besides these RNA processing and decay pathways, other RNA degrading enzymes were found to be differentially regulated in response to various virulence-related growth conditions *in vitro* and during infection *in vivo*, indicating that more RNases may play a role in mRNA stability regulation. The exploitation of advanced RNA-seq combined with new target search approaches by coupling a UV crosslinking reaction to covalently link the RNase of interest to its target RNA will elucidate the role of RNases in various cellular processes (Waters et al., 2017). Determination of the cleavage sites and profiling of cleavage products of RNases, as established for other pathogens (Linder et al., 2014; Pobre and Arraiano, 2015; Redko et al., 2016; Chao et al., 2017; Le Rhun et al., 2017), will enable us to obtain genome-wide cleavage maps of different RNases of yersiniae under virulence-relevant conditions and will facilitate unraveling their complex RNA degradation network.

## Outlook: RNA interactome and regulatory networks in *Yersinia*

The discovery of novel RNA-based regulatory strategies anticipates many exciting new insights into the complex post-transcriptional control network of virulence gene expression. Yet, determining the individual targets and interaction partners (protein and RNA) of the multitude of mRNAs, asRNAs, and sRNAs identified in *Y. pseudotuberculosis* during recent RNA-seq approaches is a time-consuming and very labor-intensive task. However, new approaches have been successfully established to generate a snapshot of the interactome of RNAs that bind to a particular RNA-binding protein, e.g., CsrA, Hfq, or YopD. Such RNA interactomes can be obtained by RNA immunoprecipitation followed by RNA-seq (RIP-seq) (Figure 2), by covalently linked protein-RNA interactions followed by RNA-seq (CLIP-seq) (Figure 2), RNA interaction by ligation and sequencing (RIL-seq), or cross-linking, ligation and sequencing of hybrids (CLASH) (Bilusic et al., 2014; Holmqvist et al., 2016; Melamed et al., 2016; Waters et al., 2017). These techniques cannot only be used to profile the individual targets and entire binding sites (positions and sequences) of an RNA-binding protein, they will also allow us to improve sRNA target prediction, and facilitate the discovery of new sRNA control circuits and regulatory pathways (Dugar et al., 2016; Heidrich et al., 2017; Michaux et al., 2017). Similarly, RNA-RNA hybrids can be enriched or cross-linked and then sequenced to obtain a genome-wide *Yersinia* RNA-RNA network as done for *E. coli* (Liu et al., 2017).

Other open questions concerning the RNA-based control network of *Yersinia* virulence factors are: How are they conserved or remodeled during evolution between different strains and species, and how do acquired variations change virulence gene expression and pathogenicity? Numerous species- and strain-specific sensory and regulatory RNAs and many intra-species variations of certain sRNAs have been discovered in human pathogenic *Yersiniae* over the past years. For instance the PhoP regulator, controlling expression of the CsrC sRNA is not expressed in *Y. pseudotuberculosis* strain YPIII, but in IP32953 (Pisano et al., 2014). Moreover, the stability of the CsrC RNAs of strain YPIII and IP32953 differs significantly due to a 20 nt insertion in CsrC of IP32953, which renders the transcript more susceptible to degradation (Nuss et al., 2014). Based on the fact that the Csr system is crucial for T3SS/Yop expression and virulence (Bücker et al., 2014; Nuss et al., 2017a), it seems likely that even small variations (patho-specific alterations) between closely related strains could have a significant influence on the pathogen's potential to readjust and adapt to different hosts, host niches and reservoirs.

Another future task will be to elucidate the precise molecular mechanism how the RNA-binding translocator protein YopD controls translation of *yop* mRNAs. One technique to investigate ribosome-associated regulation, is ribosome profiling (Ribo-seq, Figure 1). With this method, all *in vivo* positions of extracted ribosomes on mRNAs (polysomes) can be identified enabling a dynamic view of ribosome movement along all mRNAs of a bacterial cell at a certain growth condition (Li et al., 2012; Ingolia, 2014). The function of ribosome subpopulations, such as those bound by YopD could be investigated by crosslinking of the ribosomes to the mRNA followed by affinity purification of the resulting YopD-ribosomal complex.

Ongoing characterization of the identified RNA-based control mechanisms of *Yersini*a will also have to address how the different riboregulators and RNAs contribute to the colonization of different host niches and whether and how they are implicated in the reprogramming of the pathogen, e.g., *Y. pseudotuberculosis* during the switch from the acute to the persistent infection mode. As regulatory RNAs, such as CsrB and CsrC, are implicated in the control of the regulator RovA, which forms a bistable switch, leading to high- and low-invasive subpopulations (Nuss et al., 2016), it is important to investigate the impact of riboregulators and non-coding RNAs on the formation of heterogeneous subpopulation with different virulence-relevant features. A major advance for this endeavor would be the development of single-cell RNA-seq approaches, in which the transcriptome of multiple individual bacterial cells could be profiled in different host niches and hosts during the entire course of an infection. So far single-cell RNA-seq has mainly been used for eukaryotic cells, however when applied to single bacteria this approach still suffers from technical limitations, which will have to be overcome in the future. This includes the establishment of more robust analyses and a more effective single-cell lysis and cDNA synthesis (Zhang et al., 2018). The next challenge will then be to analyze and compare the huge amount of generated data in an optimized and standardized integrative manner. This demands the establishment of (i) high quality, and free accessible standardized data-bases and (ii) the development of innovative bioinformatics protocols for data analysis and interpretation that allow integration of different technical and experimental approaches (e.g., different RNA-seq techniques, different OMICs, strains, tissues, and time-points of infection). Once achieved, this will give a comprehensive view on the complex RNA biology of *Yersinia* and will open up a new level of our understanding of virulence control of bacterial pathogens.

## Author contributions

All authors listed have made a substantial, direct and intellectual contribution to the work, and approved it for publication.

### Conflict of interest statement

The authors declare that the research was conducted in the absence of any commercial or financial relationships that could be construed as a potential conflict of interest.
